# Human Papillomavirus (HPV) Prevalence in Nasal and Antrochoanal Polyps and Association with Clinical Data

**DOI:** 10.1371/journal.pone.0141722

**Published:** 2015-10-28

**Authors:** Mareike Knör, Konstantin Tziridis, Abbas Agaimy, Johannes Zenk, Olaf Wendler

**Affiliations:** 1 Experimental Otorhinolaryngology, ENT-Hospital, Head and Neck Surgery, Friedrich-Alexander University Erlangen-Nürnberg (FAU), Erlangen, Germany; 2 Department of Pathology, Friedrich-Alexander University Erlangen-Nürnberg (FAU), Erlangen, Germany; Brigham and Women's Hospital/Harvard Medical School, UNITED STATES

## Abstract

**Objectives:**

The pathogenesis of sinonasal polyposis remains unclear, in spite of several investigative approaches. Antrochoanal polyps, a subgroup of sinonasal polyposis along with allergic- and chronic-inflammatory nasal polyps, mostly originate from the maxillary sinus and develop as a unilateral, pedunculated mass towards the nasopharynx. The human papillomavirus (HPV) is discussed as a possible causative and influencing factor in development and progression of sinonasal polyposis. This study aims to elucidate HPV frequency in nasal polyps and antrochoanal polyps.

**Materials and Methods:**

Genomic DNA from 257 tissue specimens (166 nasal polyps, 39 antrochoanal polyps and 52 nasal turbinates) was subjected to three different established HPV- polymerase chain reaction assays, testing for 37 low- and high-risk HPV. In addition, immunohistochemical analyses for HPV16 were carried out, as well as immunohistochemistry and western blots of p16, a biomarker for HPV induced cancer.

**Results:**

HPV-DNA was detected in 53.8% of antrochoanal polyps, 15.1% of nasal polyps, and 5.8% of nasal turbinates. HPV16 was the predominant type with a detection rate of 76% in nasal polyps and 62% in antrochoanal polyps. Immunohistochemically, HPV positive tissues stained positive for HPV16 antigens and p16 in epithelial cell layers. No significant p16 overexpression was traceable in antrochoanal polyps, nasal polyps and nasal turbinates by western blot. There was no correlation of HPV-status with sex, age, smoking, alcohol consumption or allergic background.

**Conclusion:**

The present study shows a significant frequency of high-risk type HPV16 in antrochoanal polyps. Absence of oncogenic transformation or correlation of the HPV-status with clinical data suggests a latent superinfection, possibly because of anatomical proximity to the oropharynx.

## Introduction

Polyposis nasi et sinuum is a common disease affecting 1–4% of the general population [1–3]. It is defined as outgrowths of nasal cavity and paranasal sinus mucosa caused by allergic or chronic inflammation [4]. Generally, these outgrowths are considered to encompass predominant allergic- and chronic-inflammatory nasal polyps, here summarized as inflammatory nasal polyps. Antrochoanal polyps represent with 3–6% a minority of all nasal polyps [5, 6]. Due to this low prevalence they have so far not been studied extensively.

Nasal polyps are characterised as multifocal polypoid mucosal swellings, typical bilateral and arising mainly from the middle meatus and ethmoid sinus region. They occur more frequently in patients with persistent asthma, aspirin-exacerbated respiratory disease or cystic fibrosis and often show an allergic background [2, 4]. Furthermore, they are regularly associated with chronic sinus infection [7, 8]. The average age of patients with NP is 50 [9, 10]. Their histopathological structure is composed of stromal and subepithelial oedema as well as mixed inflammatory cell infiltration, of which more than 60% are eosinophils, along with neutrophils, mast cells and activated T- cells [1, 11]. The respiratory epithelium is usually intact and exhibits frequently goblet cell hyperplasia.

In contrast, characteristic antrochoanal polyps usually constitute a larger solitary unilateral mass. They originate from the maxillary sinus, pass through the nasal cavity, extend into the choanae and further into the nasopharynx. Antrochoanal polyps consist of two components, a cystic antral and a solid polypoid part, linked by a long stalk. Patients suffering from antrochoanal polyps are more commonly children and young adults, with an average age of 27 in our cohort and other studies [9, 10]. There is a lack of other risk factors or associated diseases. It is noteworthy that allergic diseases seem to play no significant role in the pathogenesis of antrochoanal polyps [12–14]. Accordingly they do not show morphological features typical for allergy-associated inflammatory nasal polyps [12]. Antrochoanal polyps are histologically characterized by firm fibrotic stroma showing high vascularisation with degenerative endothelial cells and stromal multinucleated giant cells [15]. They are mainly infiltrated with plasma cells and less eosinophils [15–17]. Compared to nasal polyps, antrochoanal polyps present no goblet cell hyperplasia though alterations of the ciliated epithelium are common. Even squamous cell metaplasia may be seen in antrochoanal polyps [15, 18].

Although sinonasal polyposis is a well-investigated entity, the aetiology still remains unclear. To date, quite a few studies were carried out screening nasal polyps for causative factors, including human papillomavirus (HPV) [19–23]. HPV infections are related to the genesis of various benign and malignant human neoplastic diseases, for example cervical cancer [24, 25]. Likewise a subgroup of oropharyngeal cancer is increasingly recognized to be caused by a persistent infection with high risk HPV [26, 27].

Between 2000 and 2014 only six studies investigating the association of HPV with nasal polyps and sinonasal mucosa have been published. The results are highly inconsistent and showed prevalence of HPV-DNA in nasal polyps ranging from 0% to 40%, while no viral DNA was found in healthy controls [21, 22, 28–31].

Obviously data concerning the prevalence of HPV in sinonasal polyposis is lacking. Previous studies have not investigated subgroups separately, so no references exist about a possible association of HPV in particular with antrochoanal polyps. Therefore, the current study investigated the HPV frequency in a large series of inflammatory nasal polyps and antrochoanal polyps via well-established HPV-polymerase chain reaction (PCR) arrays. To provide further information about the localisation and activity of viral infection HPV 16 and p16, a biomarker for HPV induced cancer, immunohistochemistry, as well as p16 western blot was performed.

## Material and Methods

### Tissue samples

166 archived fresh frozen specimens of nasal polyps from 2008 to 2012 were analysed. Overall, antrochoanal polyps were obtained from 39 patients, between 2000 and 2012. 31 of these were paraffin-embedded archive material from the Institute of Pathology, Friedrich-Alexander University of Erlangen-Nürnberg. Sixteen fresh frozen antrochoanal polyps were collected from patients who underwent surgical treatment at ENT-Hospital, Head and Neck Surgery, University of Erlangen-Nürnberg. There was an overlap of eight antrochoanal polyps which were available as fresh frozen as well as paraffin-embedded tissue (S1 Table, Antrochoanal Polyps). Of eight patients fresh frozen as well as paraffin-embedded tissue was available. Fifty-two fresh frozen nasal turbinates specimens with clinically normal mucosa served as control tissues. Demographic data such as sex, age, smoking/alcohol and allergic diseases was collected. Alcohol consumption and smoking was categorized as yes, if current daily, and as no, if occasionally, never or former. The Ethics Committee of the University of Erlangen-Nürnberg approved the utilisation of patient tissue for research purposes (No. 3201 and 4433). All participants were informed and gave their written informed consent.

### DNA extraction

Genomic DNA (gDNA) from paraffin-embedded tissue was extracted by using peqGOLD Tissue DNA Mini Kit (C-Line) provided by peqlab (Erlangen, Germany) following the manufacturer’s protocol. DNA isolation and purification of cryopreserved tissue was conducted with AquaGenomic^™^ reagent provided by MoBiTec GmbH (Göttingen, Germany) according to the manufacturer’s instructions. DNA concentration was evaluated using standard photometer and afterwards adjusted to 50–200 ng/μl.

### DNA detection and HPV-typing

The PCR assays used were three broad spectrum primer sets, namely established GP5+/6+ assay, short PCR fragment (SPF) PCR and E6 Nested Multiplex PCR (NMPCR) [32–35]. Furthermore type specific primers for HPV11 and HPV16 were applied [36]. The GP5+/6+ PCR were used for HPV-detection in frozen and paraffin-embedded tissues. The SPF PCR Assay was used especially for paraffin-embedded tissues. In addition, NMPCR was performed on gDNA extracted from frozen tissue. It consists of a primary amplification with a set of broad spectrum E6/7 primer, followed by nested- PCR with four cocktails of type specific primers, added together aiming for 18 HPV types [35]. Samples positive for GP5+/6+-, SPF- or NM-PCR were additionally evaluated with type specific primers for HPV11 and HPV16 [36]. To assess the quality of DNA PCR targeting β2-microglobulin gene was amplified (sense 5’-TCCAACATCAACATCTTGGT-3’, antisense 5’-TCCCCCAAATTCTAAGCAGA-3). For NM- and SPF-PCR Qiagen HotStarTaq Master Mix Kit (Qiagen, Hilden Germany) and the qPCR Core Kit SYBR^®^ Green I No ROX (Eurogentec, Cologne Germany) were used. PCRs were implemented with the iCycler (Biorad, München, Germany). Distilled water and DNA from reliable HPV negative tissue served as negative controls. Three HPV- positive oropharyngeal carcinomas were included as positive controls. The amplicons were detected by gel electrophoresis on 2.5% agarose gel and analysed with the Lumi-Imager (Roche Applied Science, Mannheim, Germany). HPV type identification was pursued by direct sequencing analysis conducted by LGC Genomics GmbH (Berlin, Germany) with primer GP6+ [33]. The sequence-evaluation was performed with BLASTN program (NCBI).

### Immunohistochemistry (IHC)

IHC on paraffin embedded sections was carried out with ImmPRESS^™^ Universal Antibody Kit (Vector Laboratories, Inc., Burlingame, USA). To make the epitopes available for antibody binding the sections underwent deparaffinization and heat-mediated antigen retrieval, using Vector^®^ Antigen Unmasking Solution pH 6 (Vector Laboratories, Inc., Burlingame, USA) at 95°C for 20 minutes. The reduction of background staining was achieved by covering the sections for 10 minutes with BLOXALL^™^ Endogenous Peroxidase and Alkaline Phosphatase Blocking Solution (Vector Laboratories, Inc., Burlingame, USA), followed by protein block with 2.5% horse serum. The antibodies HPV16 E6/18 E6 (C1P5) monoclonal mouse antibody (Santa Cruz Biotechnology, Inc., Heidelberg, Germany) and p16 (JC8) monoclonal mouse antibody (Santa Cruz Biotechnology, Inc., Heidelberg, Germany) were applied, both additionally in Pierce^®^ Immunostain Enhancer (Thermo Fisher Scientific, Bonn, Germany) and incubated over night at 4°C. A nonspecific antibody (Cell Signaling Technology, Inc., Danvers, USA) served as negative control. Afterwards the universal secondary antibody reagent was applied. Antigens were stained with Chromogen LinRed HRP-Substrat (Linaris, Dossenheim, Germany). Counterstaining was performed with haemalaun. The sections were covered with Aquatex^®^ (Merck KGaA, Darmstadt, Germany).

### Western Blots

The homogenisation (T25 Basic, IKA Labortechnik, Staufen, Germany) of 30–50 mg of each tissue sample was accomplished in 1.0 mL lysis buffer [50 mmol/L Tris, pH 7.4; 300 mmol/L NaCl; 10 mmol/L EDTA; 1% NP40; 0.1% Triton-X100; 0.1% SDS; protease inhibitor cocktail Complete^®^ (Roche, Mannheim, Germany)]. Afterwards they were incubated at 4°C for 2 hours and centrifuged at 16000g for 1 hour. The total protein concentration was determined with bicinchoninic acid assays (Thermo Fisher Scientific, Bonn, Germany). After denaturation through 5 minutes at 90°C with SDS loading buffer including mercaptoethanol, 50 μg of lysate was applied on 12% Criterion^™^ XT Bis-Tris Precast Gel (Bio-Rad, München, Germany) and transferred to nitrocellulose membranes (Protran-BA-83, Schleicher & Schuell). The blotting control was Ponceau-S stained and immunostaining of β-Actin reference protein (monoclonal rabbit antibody against β-Actin, clone 13E5, New England Biolabs GmbH Frankfurt, Germany) served as control for the loading amount of protein. The primary antibody for detection was the monoclonal mouse antibody against p16 (clone JC8, Santa Cruz Biotechnology, Inc., Heidelberg, Germany), followed by the secondary antibody, a peroxidase-labeled anti-mouse IgG, F(ab’)2 antibody (KPL, Gaithersburg, USA). The blot was incubated with SuperSignal^®^ West Dura Extended Duration Substrate (Thermo Fisher Scientific, Bonn, Germany) and the signals were detected with the Lumi-Imager (Roche Applied Science, Mannheim, Germany).

### Statistical analysis

To reveal differences between subject attributes or clinical data, as sex, smoking, alcohol consumption, allergic background and HPV- status, these variables were analysed by non-parametric Kruskal-Wallis analysis with post hoc multiple comparison rank analysis, non-parametric Mann-Whitney U test and chi-square test, as required. The statistical analysis was implemented using STATISTICA provided by StatSoft^®^ GmbH (Hamburg, Germany). P-values less than 0.05 were considered statistically significant.

## Results

### 53.8% HPV-positive antrochoanal polyps

**Fig 1:**
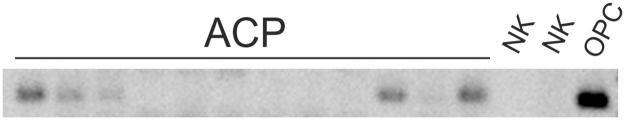
GP5+/6+ PCR. Representative extraction of broad spectrum PCR with GP5+/6+ primer. Three of twelve antrochoanal polyps (ACP) are showing clear bands, and three further weak bands. No bands are visible in negative controls (NK) and the most intense band is present in oropharyngeal carcinoma (OPC) as positive control.

In all 257 obtained tissue specimens (166 nasal polyps, 39 antrochoanal polyps and 52 nasal turbinates) gDNA was extracted and studied by broad spectrum PCR assays GP5+/6+, SPF and NMPCR, relative to tissue fixation method (see details in material and methods). Representative findings of GP5+/6+ results are depicted in Fig 1.

Overall HPV-DNA presence was detected in 15.1% of nasal polyps, 53.8% of antrochoanal polyps and 5.8% of nasal turbinates and therefore considered HPV positive [Table 1]. High significance (p < 0.001) was demonstrated in statistical analysis for 5.8% HPV-positive control tissues, as well as 15.1% HPV-positive nasal polyps in comparison to 53.5% HPV-positive antrochoanal polyps. No statistical significance was detected between nasal polyps and nasal turbinates (p = 0.08). The genotype distribution found in nasal polyps was high risk type HPV16 in 76%. In addition, 8% high risk type HPV56, 8% low risk type HPV11 and 8% HPV11/16 co-infection was found. Within the HPV- positive antrochoanal polyps, the predominant type was HPV 16 in 61.9%, followed by HPV11/16 co-infections in 23.8% and HPV11 single infection in 14.3%. Identified HPV genotypes in nasal turbinate specimens were dead level HPV16, HPV11 and HPV11/16 co-infection. Based on according results of HPV-detection in specimens of antrochoanal polyp where paraffin-embedded as well as cryopreserved tissue was available the results are not separately listed according to tissue fixation method. For further information, see supplementary data (S1 Table. Antrochoanal Polyps).

**Table 1:** HPV presence.

HPV-type	risk-type	ACP		NP		NT	
		Total (n = 39)		Total (n = 166)		Total (n = 52)	
		N	%	N	%	N	%
Total		21	53.8	25	15.1	3	5.8
**11**	low	3	14.3	2	8	1	33.3
**16**	high	13	61.9	19	76	1	33.3
**11/16**	low/high	5	23.8	2	8	1	33.3
**56**	high	0	0	2	8	0	0

Table 1 shows the number of all obtained tissue specimens and exact number and percentage of HPV- positive samples of antrochoanal polyps (ACP), nasal polyps (NP) and nasal turbinates (NT). Furthermore, it shows the exact number and percentage of detected HPV types for each tissue group.

These findings support the assumption that antrochoanal polyps constitute a distinct subgroup of nasal polyps. In contrast, the majority of sinonasal polyposis, i.e. nasal polyps does not show a significant higher HPV-association than control tissue, pointing out that there is no aetiological association.

### Positive staining for HPV 16 E6 protein in epithelial cell layers of HPV-positive nasal- and antrochoanal polyps

**Fig 2:**
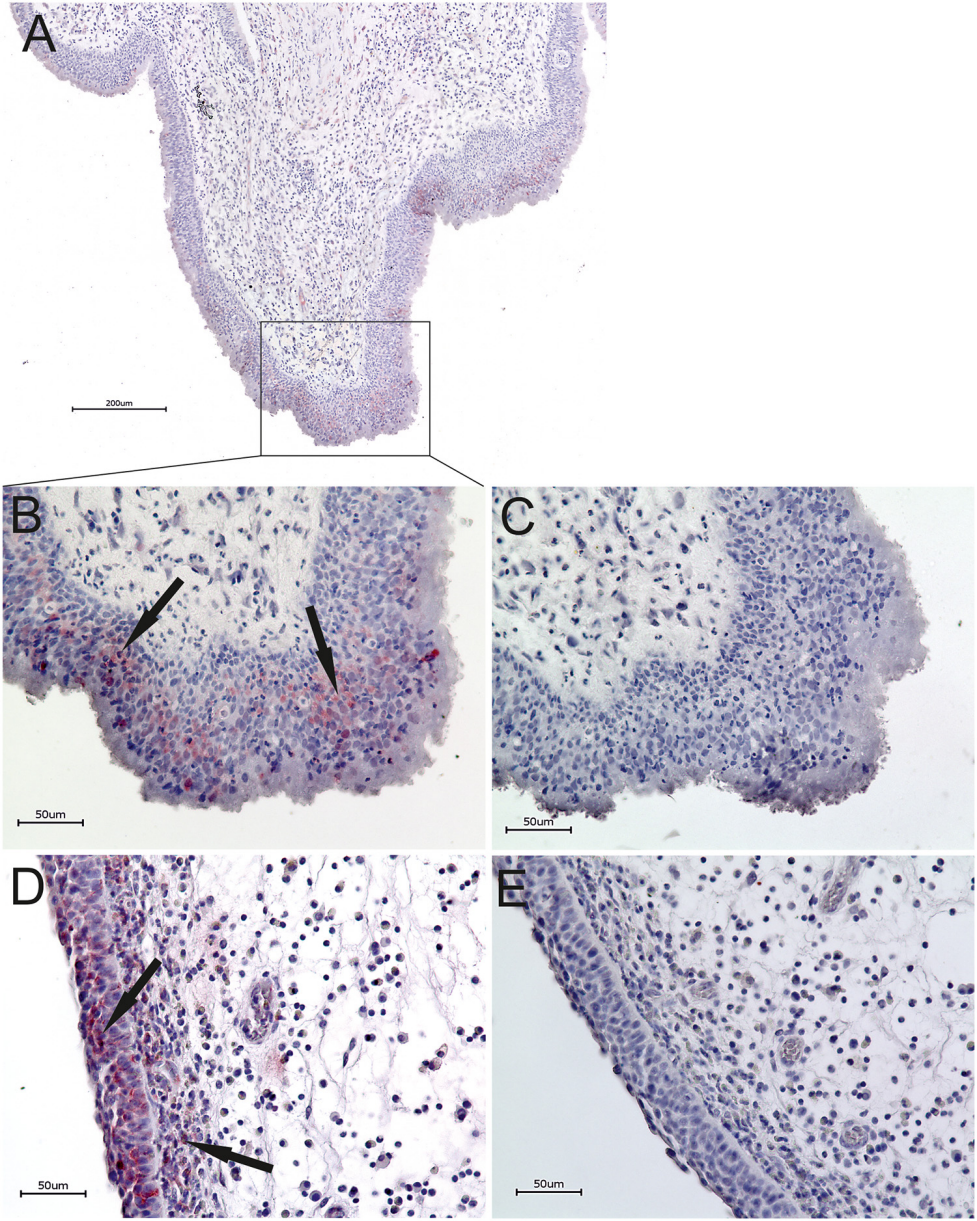
HPV Immunohistochemistry. IHC Overview of a representative HPV-positive antrochoanal polyp with HPV16 staining (A). HPV16 E6 protein staining of a representative HPV-positive antrochoanal polyp, relevant epithelial region shown (B) with corresponding negative control (C). HPV16 E6 protein staining of a representative HPV-positive nasal polyp, relevant epithelial region shown and subepithelial stained cells (D) with corresponding negative control (E). Discontinuous epithelial cell clusters stained positive in HPV-positive antrochoanal- and nasal polyps.

To locate the virus HPV 16 E6 protein immunohistochemistry of HPV-positive nasal- and antrochoanal polyps was performed. In all presented cases discontinuous cell clusters stained positive, limited to the epithelium (Fig 2A). In antrochoanal polyps the stained regions were mostly apparent in epithelial sections showing epithelial changes, such as stratification. The signals were located in nuclei and cytoplasm. Within the epithelium the signals frequently occurred in middle and apical cell layers, seldom in basal cell layers. The stained regions were mostly apparent in epithelial sections also showing epithelial changes, as stratification and ciliumdegeneration. The stroma consisted of firm and fibrotic tissue and showed minor inflammatory cell infiltration (Fig 2B and 2C). In nasal polyps cytoplasmatic and intranuclear signals were detected in all cell layers, without clear accentuation. In the stained regions no signs of epithelial stratification or degeneration could be found. Beyond the epithelial signals also cells near to the basement membrane in the oedematous stroma stained HPV16 positive. There was an obvious subepithelial infiltration of inflammatory cells in NP (Fig 2D and 2E). It is noteworthy that the signals found in antrochoanal- and nasal polyps were not detectable using standard staining procedure without immunostain enhancer, indicating a very low level of E6 protein.

### P16 immunohistochemistry shows weak signals in epithelial layers of HPV-positive antrochoanal polyps

**Fig 3:**
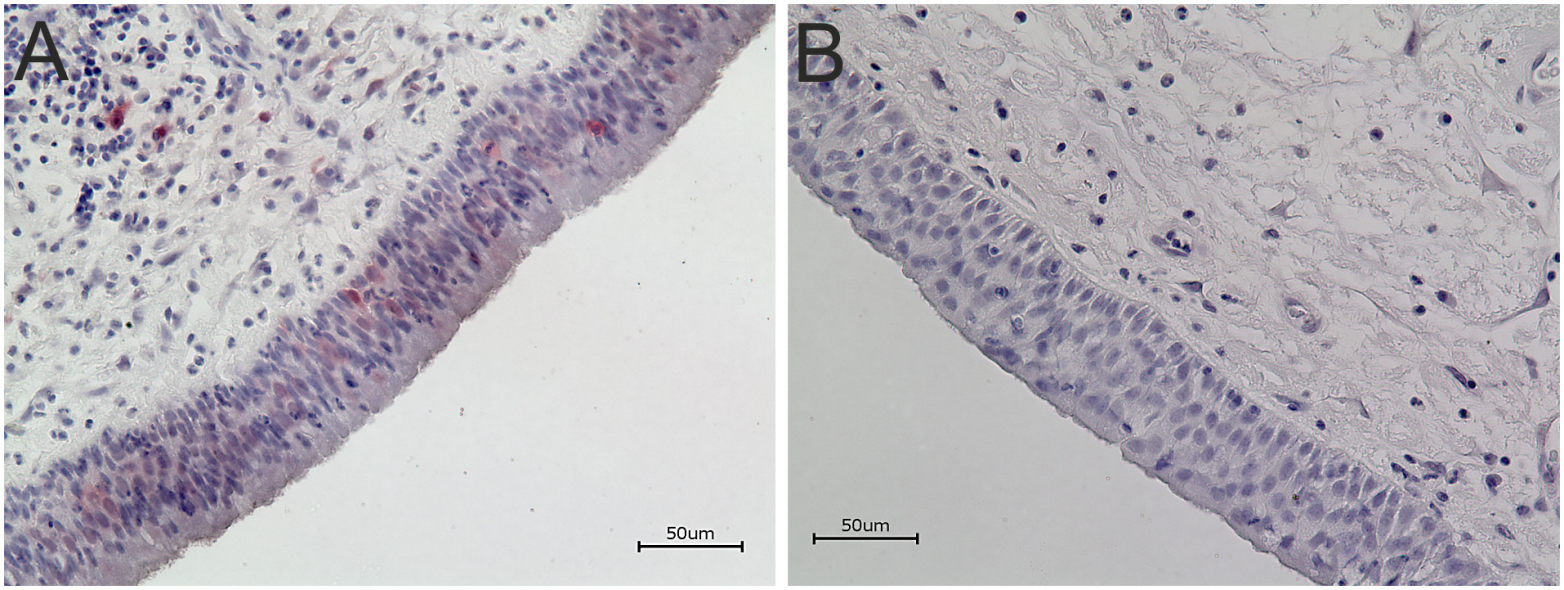
P16 Immunohistochemistry. Immunohistochemistry of p16 protein expression in representative HPV-positive antrochoanal polyp (A) and corresponding negative control (B). Weak irregular spread epithelial p16-IHC staining in strong HPV-DNA positive samples. Detected signals were dominantly intranuclear.

To evaluate p16 expression of HPV-positive and -negative nasal- and antrochoanal polyps, p16-IHC was performed, also with enhancer staining. The p16-IHC showed a similar sectional staining in HPV-DNA positive samples as we have seen in HPV16-E6-IHC. Compared to HPV16-E6 staining the signals were weaker and irregularly distributed within the epithelium but unequivocally identifiable. The detected signals were predominantly intranuclear (Fig 3). It has to be mentioned that p16 staining was only positive in five cases of antrochoanal polyps, with strong HPV-DNA positivity. In nasal polyps and nasal turbinates p16-IHC was negative.

### In western blot p16 is traceable without significant increase in all tissue specimens

**Fig 4:**
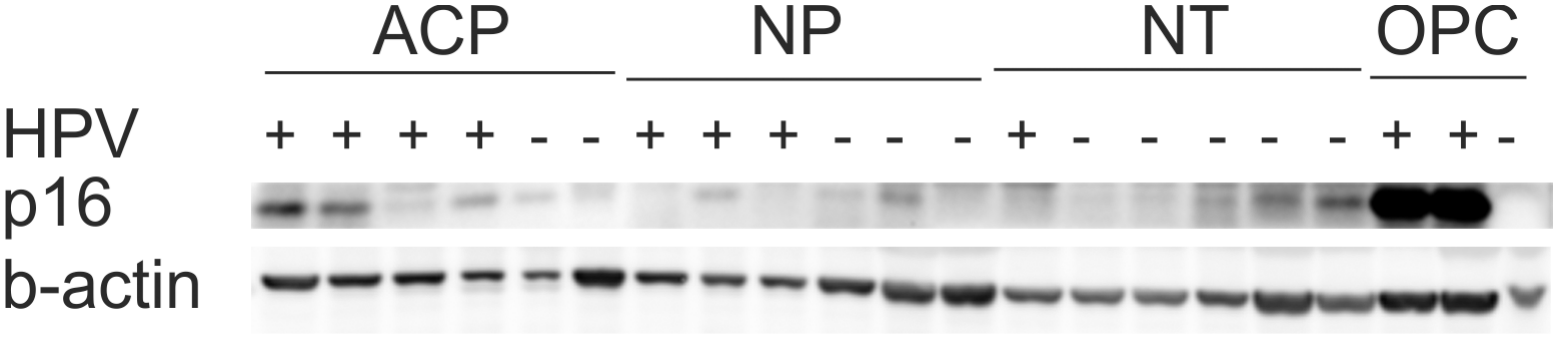
P16 Western Blot. Representative p16 western blot of HPV-positive and -negative antrochoanal polyps (ACP), nasal polyps (NP), nasal turbinates (NT) and oropharyngeal carcinoma (OPC). Two HPV-positive carcinomas are showing strong p16 bands. Three of four HPV-positive.

The analysis of the cellular protein p16 with western blot was performed additionally to immunohistochemistry of HPV-positive and -negative nasal- and antrochoanal polyps in comparison to negative control tissue and oropharyngeal carcinomas. It was performed because of the slight signal strength revealed in IHC and because it is giving a hint about p16 amount. HPV-positive carcinomas showed the most intense bands pointing out a highly increased p16 expression, also shown by Hoffmann, M., et al., 2010 [37]. In contrast no p16 bands were visible in HPV-negative carcinomas. Weak bands were traceable in HPV-positive antrochoanal polyps, indicating a slightly elevated p16 expression in strong HPV-positive antrochoanal polyps. Nasal polyps and nasal turbinates showed occasional weak bands in HPV-negative and positive cases. Altogether the detected p16 expression in nasal polyps, antrochoanal polyps and nasal turbinates seems to be on a regular level. The loading control with β-Actin reference protein was mainly comparable in all lines (Fig 4).

### Association of HPV status and demographic data

Our clinical series was composed of 166 nasal polyps, 39 antrochoanal polyps and 52 nasal turbinates as control tissue. The statistical analysis referred to the collected characteristics HPV- status, sex, age, smoking, alcohol consumption and allergic background, inter- and intra-group [Table 2].

**Table 2:** Clinical data.

	HPV-PCR	ACP		NP		NT	
		Total (n = 39)		Total (n = 166)		Total (n = 52)	
		N	%	N	%	N	%
Total	**-**	18	46.2	141	84.9	49	94.2
	**+**	21	53.8	25	15.1	3	5.8
Age							
	**-**	29	-	46	-	33	-
	**+**	23	-	50	-	49	-
Gender							
male	**-**	7	17.9	89	53.6	34	65.4
	**+**	13	33.3	17	10.2	2	3.8
female	**-**	11	28.2	52	31.3	15	28.8
	**+**	8	20.5	8	4.8	1	1.9
Allergy							
yes	**-**	7	17.9	83	50	19	36.5
	**+**	7	17.9	11	6.6	2	3.8
no	**-**	11	28.2	58	34.9	30	57.7
	**+**	14	35.9	14	8.4	1	1.9
Smoking							
yes	**-**	3	7.7	49	29.5	23	44.2
	**+**	2	5.1	10	6	0	0
no	**-**	15	38.5	92	55.4	26	50
	**+**	19	48.7	15	9	3	5.8

Table 2 shows the demographic data and characteristics of nasal polyps (NP), antrochoanal polyps (ACP) and nasal turbinates (NT) used in the current study. Alcohol consumption closely resembles Smoking.

**Fig 5:**
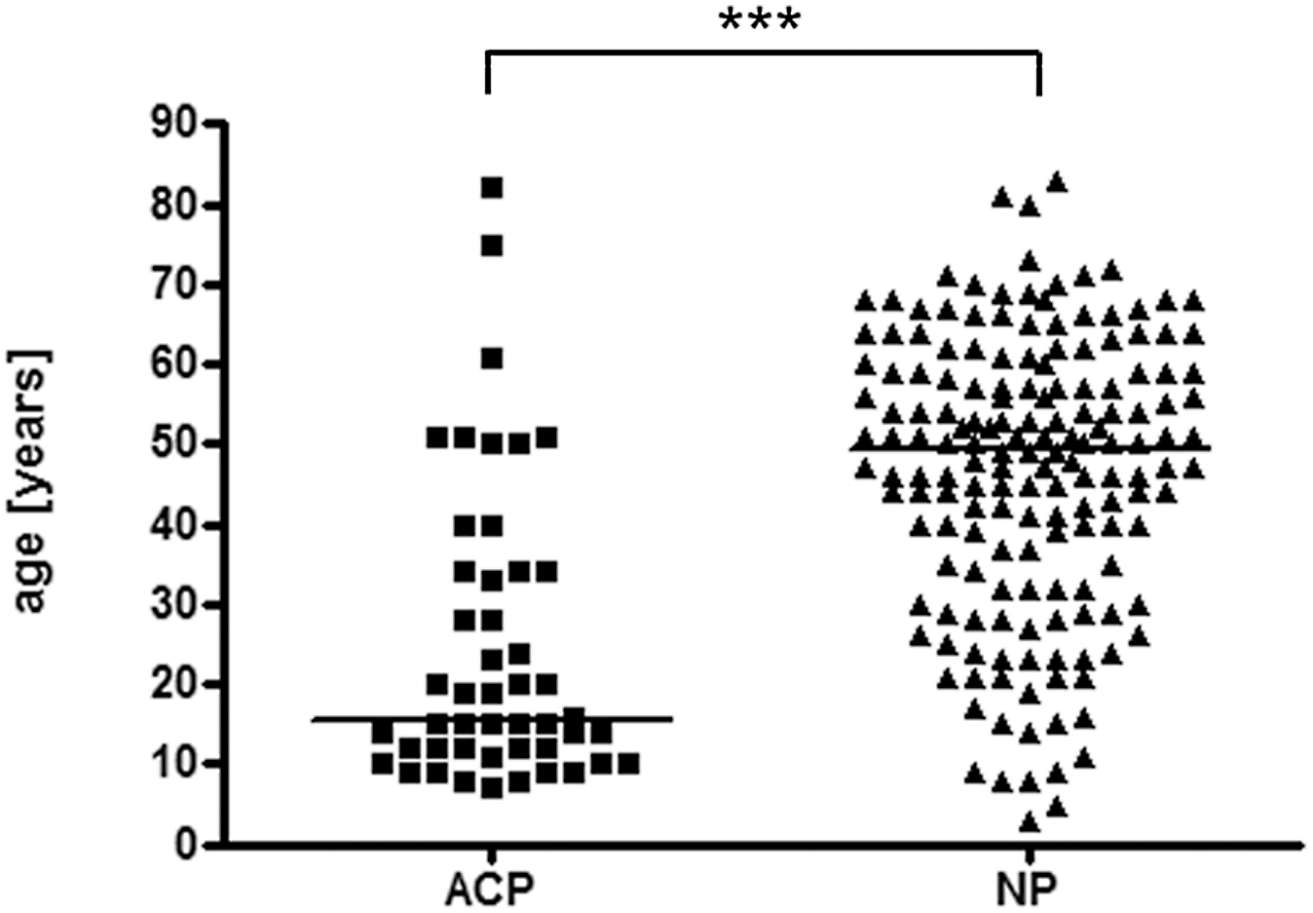
Age distribution of antrochoanal- and nasal polyps. The Boxplot shows high significant difference in age distribution of antrochoanal polyps (ACP) in comparison to nasal polyps (NP). Asterisks symbolize significance levels of multiple comparison rank analysis (* p < 0.05; ** p < 0.01; *** p < 0.001).

The male-female ratio in nasal polyps was nearly 2:1 and in antrochoanal polyps 1:1, but without statistically significant difference. Non-parametric Kruskal-Wallis ANOVA analysis of age distribution showed statistical significance with H = 48.07 and p < 0.0001. Post hoc multiple comparison rank analysis demonstrated significant differences of age among nasal- and antrochoanal polyps, as well as among nasal polyps and nasal turbinates (Fig 5). Analysis of age distribution restricted to HPV positive or negative cases revealed no statistically significant changes between and within the groups. Among the multivariate statistical evaluation of smoking, alcohol consumption and allergic background only alcohol consumption showed a statistically significant difference (p < 0.016) between nasal- and antrochoanal polyps, but this may be biased by the lower mean age of patients with antrochoanal polyps. The multivariate analysis of these three clinical parameters relative to HPV- status revealed no further differences (p = 0.05). After statistical analysis none of these data could be correlated with the HPV status.

## Discussion

The major purpose of this study was to further investigate the HPV-prevalence in nasal polyps and specially the subgroup of antrochoanal polyps, revealing unexpected significant differences.

Including 166 fresh frozen nasal polyps, 39 antrochoanal polyps and 52 nasal turbinates this study represents one of the largest published on this topic. The number of evaluated nasal polyp specimens in previous studies has been usually in a range of 20–48 [21, 22, 28, 30, 31], except one Chinese publication with 204 paraffin-embedded specimens [29]. In most studies 10–36 cases of normal mucosa as controls were included, whereas one enclosed 85 hypertrophied turbinates [21, 22, 28–31].

Overall, the available published data reporting on HPV-DNA presence in nasal polyps have been inconclusive, ranging from 0% to 50% HPV-frequency in NP [21, 22, 28–31]. Two Chinese studies recorded 0% and 40% HPV- prevalence, respectively [28, 29]. Out of four European groups, two German studies failed to detect HPV in nasal polyps [21, 22], one Italian group reported 50% HPV positive cases in 20 samples [31] and the results from a Greek group closely resembles our findings with a HPV- prevalence of 13% in nasal polyps and 4% in adjacent turbinates [30]. With 53.8% HPV-frequency in antrochoanal polyps, our study shows a significant higher association of them with HPV than inflammatory nasal polyps and nasal turbinates.

The recently reported HPV-genotypes found in nasal polyps included high- and low risk types, 75% were single infections [29]. Most prevalent was low-risk type HPV11 with 45.28%, followed by high-risk HPV58 with 16.04% and high-risk HPV52 with 10.38% [29]. Zaravinos et al, 2009, searching for HPV 6, 11, 16, 18, 33, detected neither low-risk nor high risk HPV-types. Therefore, the detection of oncogenic HPV16 clearly dominating the genotype distribution in our study was unexpected.

The frequently used method of HPV-detection was MY09/11 PCR Assay, applied to cryopreserved as well as paraffin-embedded nasal polyps [21, 22, 28], moreover GP5+/6+ PCR assay [30], HPV flow through hybridisation and gene chip method [29]. Because of varying DNA-detection methods which was used without adaptation to diverging fixation methods and to offer a maximum of certainty, we carefully selected the PCR assays. For example, in oropharyngeal carcinomas the long approved general PCR Assay GP5+/6+ has been proven more sensitive than MY09/11 PCR Assay, especially in low viral load samples [38, 39]. GP5+/6+ PCR Assay has been applied on all specimens in our study, it targets 140 bp in the L1 gene [33, 34]. In contrast to the high viral load found in HPV associated oropharyngeal cancer, there merely seems to be a low copy number in HPV-positive nasal polyps, regarding the difference in the intensity of bands (Fig 1). Additionally, consensus primer system SPF has been implemented for paraffin-embedded tissue. It has been developed with the purpose to improve the HPV detection in paraffin-embedded samples [32] and therefore amplifies a very short fragment of 65bp in the viral gene L1. To further increase the sensitivity, especially in detection of the assumed low viral load and to confirm the first results, NMPCR was carried out, which provides the advantage of simultaneous HPV type identification without sequencing [35, 40–42].

An established procedure to detect a HPV-infection causative for malignant transformation recommends p16 immunohistochemistry and HPV-DNA detection [37, 43, 44]. P16 overexpression, as a result of inactivation of pRb by E7 gene product has been proposed as surrogate marker for biologically active HPV infections in malignant neoplasia [45, 46]. It highly correlates with HPV-infection [47, 48]. Furthermore, p16 overexpression can also be alternatively induced by mechanisms otherwise than HPV interactions, as discussed in detail elsewhere [49–51]. P16-IHC here showed inconclusive results in terms of very slight, mainly intranuclear signals in the epithelium of strong HPV positive antrochoanal polyps. Subsequently p16 western blot was performed, revealing no significantly elevated p16 expression in nasal- and antrochoanal polyps in contrast to nasal turbinates. There was no oncogenic p16 upregulation as reported in HPV induced malignancies, in correspondence to strong p16 staining in IHC and intense bands in western blot [37, 45, 46], as has also been shown within the positive controls here (Fig 4). The p16-IHC-positive cases and the slightly elevated p16 amounts in western blot in antrochoanal polyps are more likely to be a result of present simultaneous inflammation [50] than of HPV induced dysregulation.

The HPV16-E6-IHC is not yet a standard method. It provides a hint about viral transcriptional activity and its localization. HPV16 E6 protein seems to be required for genome establishment and maintenance as extrachromosomal nuclear plasmid in host cells [52]. The results showed weak intranuclear and cytoplasmatic signals, pointing out that the virus is present and established in the cells with low level transcriptional activity. No high transcriptional activity leading to oncogenic transformation is assumed. But possibly epithelial changes are induced as stratification and ciliumdegeneration. The discontinuous staining of cell clusters can be seen as invasion zones. Perhaps epithelial damage facilitates the infection and viral establishment. The cells near to the basement membrane in the stroma in nasal polyps that stained HPV16 positive can probably be HPV positive epithelial cells transmigrated into the stroma or solely an artificial staining of subepithelial inflammatory cells. For further investigations staining of basement membrane would be a promising candidate method to prove its integrity.

Our data reported here are in line with findings about age and sex distribution of nasal- and antrochoanal polyps, as reported in currently available literature [9, 10]. The statistical analysis of clinicopathological data confirmed that neither age nor sex nor smoking nor alcohol consumption nor allergic background correlates with the HPV status [29, 53]. The statistically significant difference (p < 0,016) detected for alcohol consumption is highly probable a bias due to the fact that one third of antrochoanal polyp patients are children [6, 17].

Currently there is little evidence of a causal relationship of HPV infection in nasal polyps and their progression [29], especially when solely HPV DNA presence is proven with PCR and sequence analysis. To identify a biological active HPV infection the verification of viral transcriptional activity is required. The results of p16-IHC and western blot as well as HPV16-E6-IHC to prove an active infection, possibly causative for proliferation, were difficult to establish and to interpret. This is likely due the low viral load in nasal- and antrochoanal polyps compared to cervical or oropharyngeal cancer. The existing surrogate marker p16 seems to play no relevant role with respect to nasal polyps. Also HPV16-E6-IHC pointed out that there is no relevant transcriptional activity of HPV in nasal polyps. RNA detection of E6/7oncogene has been named method with high sensitivity to prove transcriptional activity in cervical and oropharyngeal cancer [54–57] An important point is, that only tumours with a high viral load seem to express E6/7 mRNA, suggesting an etiological involvement [51, 57–59]. This is also in accordance with first results of E6/7 mRNA qPCR in this study (data not shown). There was no E6/7 mRNA expression in HPV-positive nasal- and antrochoanal polyps detectable, postulating no involvement in their development.

Taken together it is difficult to compare the varying prevalence of HPV in sinonasal polyposis reported by different studies due to differences in factors such as race, geographical region, laboratory techniques used, tissue fixation method, number of samples and choice of controls. The results presented here clearly indicate the existence of an association of HPV with nasal polyps, although in a lesser extent than with antrochoanal polyps. In either case this association does not seem to initiate a proliferation or actually an oncogene transformation and is not assumed a causative factor for their development. We propose that it rather is matter of coincidental transient infection.

## Supporting Information

S1 Table Antrochoanal Polyps. Table shows all 39 antrochoanal polyps and detailed data concerning age, gender, tissue fixation method and HPV-PCR results.
(DOCX)
