# Supplementary material for: Human Papillomavirus (HPV) Prevalence in Nasal and Antrochoanal Polyps and Association with Clinical Data
Source: PLoS One. 2015 Oct 28;10(10):e0141722. doi: 10.1371/journal.pone.0141722 (PMC4624970; doi:10.1371/journal.pone.0141722)
Supplement: S1 Table — Table shows all 39 antrochoanal polyps and detailed data concerning age, gender, tissue fixation method and HPV-PCR results. (DOCX) [file pone.0141722.s001.docx]

|  |  |  |  |  |  |  |
| --- | --- | --- | --- | --- | --- | --- |
| No. | gender | age | tissue fixation paraffin fresh frozen | | HPV-type 11 16 | |
| C1 | m | 34 | + | + |  | + |
| C2 | f | 14 | + | + |  | + |
| C3 | m | 12 | + | + |  | + |
| C4 | m | 28 |  | + |  |  |
| C5 | f | 15 |  | + |  |  |
| C6 | f | 9 |  | + |  |  |
| C7 | f | 82 |  | + |  |  |
| C8 | f | 10 |  | + |  | + |
| C9 | f | 9 |  | + |  | + |
| C10 | m | 40 | + | + | + |  |
| C11 | f | 10 | + | + |  |  |
| C12 | m | 15 |  | + | + | + |
| C13 | m | 61 |  | + | + |  |
| C14 | m | 15 | + | + | + | + |
| C15 | m | 50 | + |  | + | + |
| C16 | f | 8 | + |  |  | + |
| C17 | f | 34 | + | + |  |  |
| C18 | f | 51 | + | + |  |  |
| C19 | m | 12 | + |  |  | + |
| C20 | m | 11 | + |  |  |  |
| C21 | m | 10 | + |  | + | + |
| C22 | f | 8 | + |  |  |  |
| C23 | m | 12 | + |  |  |  |
| C24 | m | 23 | + |  |  |  |
| C25 | f | 20 | + |  |  | + |
| C26 | m | 20 | + |  | + | + |
| C27 | f | 12 | + |  |  |  |
| C28 | m | 14 | + |  |  |  |
| C29 | f | 51 | + |  |  |  |
| C30 | m | 16 | + |  |  | + |
| C31 | f | 20 | + |  |  |  |
| C32 | f | 24 | + |  |  | + |
| C33 | m | 33 | + |  |  | + |
| C34 | m | 74 | + |  |  |  |
| C35 | f | 13 | + |  | + |  |
| C36 | f | 19 | + |  |  |  |
| C37 | f | 7 | + |  |  | + |
| C38 | m | 61 | + |  |  | + |
| C39 | f | 50 | + |  |  |  |
|  |  |  |  |  |  |  |
|  |  |  |  |  |  |  |
